# Screening for atypical porcine pestivirus in Swedish boar semen used for artificial insemination and a characterisation of the seminal RNA microbiome including the virome

**DOI:** 10.1186/s12917-023-03762-6

**Published:** 2023-10-20

**Authors:** Hedvig Stenberg, Maja Malmberg, Juliette Hayer

**Affiliations:** 1 Department of Biomedical Sciences and Veterinary Public Health, Swedish University of Agricultural Sciences, SLU, P.O. Box 7028, 750 07 Uppsala, Sweden; 2 MIVEGEC, University of Montpellier, IRD, CNRS, Montpellier, France

**Keywords:** Atypical porcine pestivirus, Congenital tremor, Semen, Breeding boars, Artificial insemination, Seminal, Virome, Microbiome, Pigs, High-throughput sequencing

## Abstract

**Background:**

This study aimed to characterise the RNA microbiome, including the virome of extended semen from Swedish breeding boars, with particular focus on Atypical porcine pestivirus (APPV). This neurotropic virus, associated with congenital tremor type A-II in piglets, was recently demonstrated to induce the disease through insemination with semen from infected boars.

**Results:**

From 124 Artificial Insemination (AI) doses from Swedish breeding boars, APPV was detected in one dose in addition to a sparse seminal RNA virome, characterised by retroviruses, phages, and some fecal-associated contaminants. The detected seminal microbiome was large and characterized by Gram-negative bacteria from the phylum Proteobacteria, mainly consisting of apathogenic or opportunistic bacteria. The proportion of bacteria with a pathogenic potential was low, and no antimicrobial resistance genes (ARGs) were detected in the datasets.

**Conclusion:**

Overall, the results indicate a good health status among Swedish breeding boars. The detection of APPV in semen raises the question of whether routine screening for APPV in breeding boars should be instigated.

## Background

Porcine breeding is based on artificial insemination (AI) using chilled semen with antibiotics added to the extender [1, 2]. One ejaculate may be used to provide 20 to 60 AI doses, which have rapidly improved the rate of genetic progress in pig breeding [1, 2]. While the use of AI combined with health control programmes has decreased the risk of disease transmission compared to natural service, semen for AI is still one important transmission route of infectious diseases into pig farms [3–6]. Surprisingly, little to almost nothing is known about the seminal microbiome and virome of breeding boars [7, 8].

A key example of virus that may be transmitted through AI-semen is atypical porcine pestivirus (APPV), which was recently demonstrated to have caused an outbreak of congenital tremor following introduction via AI-semen on a closed farrow-to-finish farm [9]. Congenital tremor is a neurological disorder presenting with tremor and ataxia in new-born piglets [10–13], with a mortality that may approach 20 – 50% [14, 15]. The disease occurs when a pregnant sow is infected with APPV and is mainly seen in gilt litters [12, 16]. Hence, APPV may only induce congenital tremor in piglets born to immunologically naïve, pregnant dams [11, 12, 14]. Further, the virus has not been associated with any signs of clinical disease following horizontal infection [11, 17]. Although the infection route of APPV is not yet fully understood, horizontal as well as vertical transmission are both possible [17] and long-time or persistent shedding of APPV in stool, urine, saliva, and semen from clinically healthy pigs has been reported following in-utero infections [11, 12, 18]. The virus has been demonstrated in semen samples from breeding boars in the USA [5] but, so far, APPV is to our knowledge not included in any control program for breeding boars.

The virus has been detected in domestic pigs globally [13, 16, 18–22] and in wild boars in Europe and Asia [23–27]. While the virus was first characterized in 2015 [19], it has likely caused congenital tremor in piglets described historically [14, 28]. In Sweden, on-farm outbreaks from congenital tremor have been reported for over 50 years [14], but APPV was only recently demonstrated [13, 27, 29].

Artificial insemination is the dominant breeding technique in the Swedish pig industry and is practiced in almost 100% of the farms. In 2021, the trade association for Artificial Insemination semen in Sweden (Svenska Köttföretagen AB) sold approximately 700 000 porcine AI doses. Commercial boar semen in Sweden originates from two boar studs and includes four breeds; Landrace, Yorkshire, Duroc (Norsvin & DanAvl), and Hampshire. In total 400 approved boars are on these studs at any given time. Every sixth week, 25 new boars are taken in to the quarantine for testing and evaluation. The breading boars are kept under a specific pathogen control program and are treated with anthelmintics, tested, and vaccinated against some listed pathogens However, APPV is not included in the control list.

Currently, there are no published studies on the porcine seminal virome and to our knowledge only two studies of the porcine seminal microbiome exist [7, 8]. To address these research gaps, we aimed to characterise the RNA microbiome including the virome of extended semen from Swedish breeding boars, with a particular focus on APPV.

## Results

### Metagenomic sequencing of semen pools

**Fig. 1:**
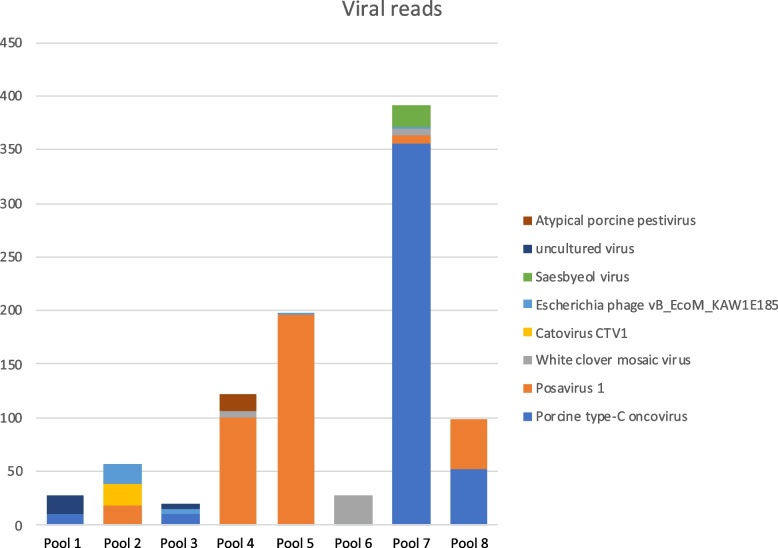
Stacked bar chart showing the number of reads from the eight most abundant viral species. Reads classified as Equine infectious anemia virus were removed from the dataset for clarity. Atypical Porcine Pestivirus generated 16 reads in pool number 4

After quality control and filtering, the sequencing of eight semen pools generated between 4.4 – 15.7 million reads per sample with a mean read length of 130 bp. Of these reads, an average of 24% mapped against the host genome (*Sus scrofa domesticus*) and were removed. Of the remaining reads, approximately 98% could be taxonomically classified. A majority of the classified reads, approximately 55%, were of microbial origin, mainly bacterial. Less than 1% of the reads were classified as viral (Table 1). The specific alignment against the APPV-genome identified APPV-sequences in one of the eight semen pools (pool 4; Fig. 1).

**Table 1:** Sequencing and assembly metrics for all 8 datasets

**Pool id**	**Number of reads before QC**	**Read length before QC**	**Number of good quality reads**	**Read length good quality reads**	**% Host mapped**	**Total nr of contigs**	**Viral reads**	**Viral contigs**	**Bacterial reads**	**Bacterial contigs**
**Pool 1**	17.3 M	151bp, 151bp	15.7 M	133bp, 133bp	20,85%	5280	16,021	7	3.2 M	1628
**Pool 2**	8.7 M	151bp, 151bp	7.2 M	125bp, 125bp	39.55%	5013	10,776	6	0.96 M	769
**Pool 3**	6.6 M	151bp, 151bp	6.1 M	122bp, 122bp	47,09%	7976	3890	4	0.63 M	883
**Pool 4**	4.8 M	151bp, 151bp	4.4 M	135bp, 135bp	13,98%	3216	6048	10	1 M	1349
**Pool 5**	6.2 M	151bp, 151bp	5.6 M	132bp, 132bp	20,59%	2810	6419	10	1.1 M	758
**Pool 6**	14.6 M	151bp, 151bp	13.4 M	131bp, 131bp	12,83%	5252	15,707	5	3 M	1418
**Pool 7**	13.6 M	151bp, 151bp	12.7 M	128bp, 128bp	14,04%	4050	12,622	4	2.9 M	2229
**Pool 8**	7.0 M	151bp, 151bp	6.8 M	130bp, 130bp	24,43%	4621	4550	5	1.2 M	1042

At the contig level, approximately 94% of the de novo assembled contigs could be taxonomically classified. The majority of the contigs, on average 60% in each dataset, were of chordate origin, mainly *Sus scrofa domesticus*. About 35% of the contigs were of microbial origin, mainly bacterial. Less than 1% of the classified contigs were of viral origin.

The datasets generated and analysed during the current study are available in the RJEB51770 repository at the European Nucleotide Archive. [https://www.ebi.ac.uk/ena/browser/view/PRJEB51770, accession number: SAMEA13468669 — SAMEA13468676].

### Limited virus diversity in boar semen

Across the eight pools, we detected three viral species which generated > 300 reads per sample: Equine infectious anemia virus, Porcine type-C oncovirus, and Posavirus-1. Other detected viral species generated < 25 reads per pool. Atypical Porcine Pestivirus generated 16 reads in one of the pools (pool4; Fig. 1).

Reads classified as Equine infectious anemia virus were most abundant, detected in all pools, with one pool containing > 15 000 reads. When reads classified as Equine infectious anemia virus were extracted and blasted, they generated Equine infectious anemia virus genome hits. These contigs were the most common contigs, present in all pools, and generated in total 16 contigs. However, it should be noted that virus classified as Equine infectious anemia virus is commonly detected in metagenomic dataset due to reagent contamination by viruses highly similar to Equine infectious anemia virus [30] and are therefore not considered a clinically relevant finding and thus not further investigated.

The second most common viral finding was classified as a Porcine type-C oncovirus. Reads from this virus were detected in all pools (1–356 reads) with a maximum of 356 reads in one pool (pool 7; Fig. 1). When blasted, the reads generated Porcine type-C oncovirus hits, an endogenous retrovirus integrated in the genome of all pigs [31, 32], not considered clinically relevant, and is therefore not further investigated.

**Fig. 2:**
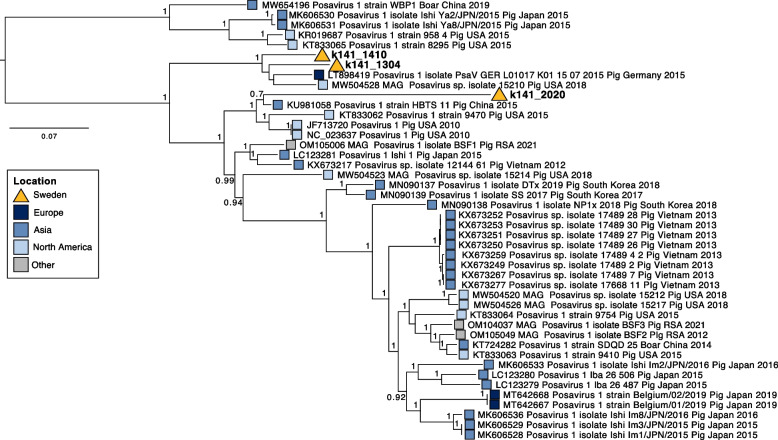
Maximum likelihood tree of Posavirus-1. Tree is midpoint rooted for clarity only, scale bar corresponds to the number of nucleotide sequences per site and aBayes branch support values are provided at nodes. Coloured boxes indicate continent of virus detection. Viral contigs recovered here are denoted in bold

Reads classified Posavirus-1, an unassigned member of the *Picornavirales*, were detected in all pools with a maximum of 194 reads in one pool (pool 5; Fig. 1), which were assembled into 14 contigs in five out of eight pools. Three contigs from pool 4, ranging from 370-634 bp, spanning the non-structural coding region (specifically the helicase), were further investigated using maximum likelihood phylogenetic analysis (Fig. 2). These sequences were 90–95.8% similar to Posavirus-1 sequences using blast, and two contigs fell into a clade comprising sequences from pigs in Germany (LT89419) and the USA (MW504528). The third contig was sister to sequences from the USA and China, but featured a long branch length.

### Atypical porcine pestivirus in the semen

**Fig. 3:**
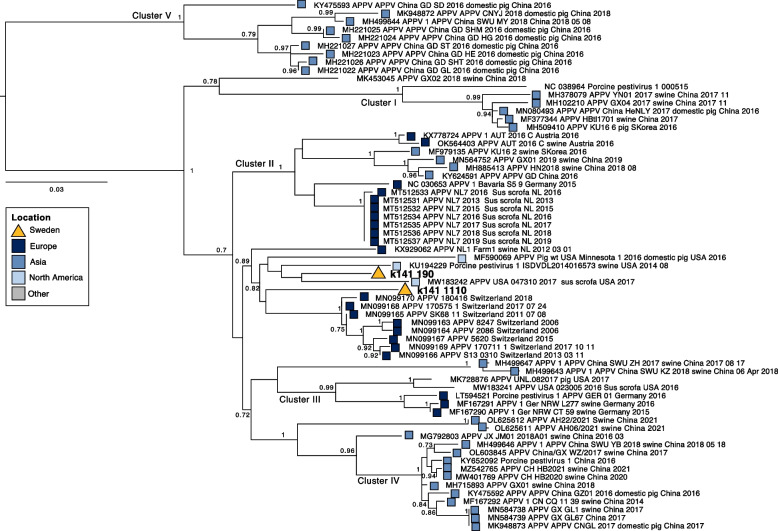
Maximum likelihood tree of Atypical Porcine Pestivirus. Cluster number as defined by Gatto et al., [35]. Tree is rooted against Cluster V viruses, as per Gatto et al., [35]. Scale bar corresponds to the number of nucleotide sequences per site and aBayes branch support values are provided at nodes. Coloured boxes indicate continent of virus detection. Viral contigs recovered here are denoted in bold

In one pool, 16 reads classified as APPV were detected. These APPV reads were also identified by the specific alignment against the APPV-genome and generated APPV-hits when blasted. Reads were assembled into two short contigs, 425 bp and 334 bp, comprising the 5’UTR and spanning the glycoproteins E^rns^ and first half of E1. These contigs were further investigated using maximum likelihood phylogenetic analysis (Fig. 3). One contig (k141_1110; Fig. 3), 425 bp long, shared a 97.57% sequence identity with an APPV-sequence (KU194229.1) retreaved from a domestic pig sampled 2016 in USA [11]. The other contig (k141_190), 334 bp, was most closely related to an APPV-sequence from a domestic pig collected in 2018 in Switzerland (MN099170.1) [33], with an identity of 95.2%. Similar to the APPV previously detected in Sweden, from piglets with signs and congenital tremor type A-II and wild boars [13, 27], the APPV obtained in the boar semen in the present study falls in to genotype III [34].

As the pools comprised 3 animals, to identify which individual boar(s) were shedding virus, a qRT- PCR specific for the NS3B region of the APPV-genome was used on the 24 semen samples individually. Of the 24 samples, the semen sample from one boar, was PCR-positive for APPV-genome (Cq-value: 28.51).

To address the frequency of APPV in boar semen, in 2021, 100 single boar AI doses were collected from Swedish Hampshire boars and screened for the presence of APPV-genome. None of these samples were PCR-positive for APPV.

### Phages

**Fig. 4:**
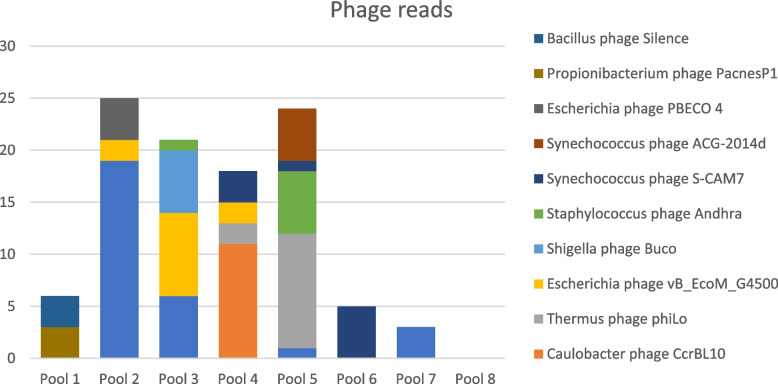
Stacked bar chart showing the number of reads from the ten most abundant phage species

In total, 121 reads classified as 20 different phages were detected in seven of eight datasets (6—28 phage reads in pool 1—7). Escherichia phage vB_EcoM_KAW1E185 was the most abundant species with a total of 29 reads detected in four out of eight datasets (pool 2, 3, 5, and 7; 1–19 reads per pool) followed by Thermus phage phiLo with a total of 13 reads detected in two out of eight datasets (pool 4 and 5; 2–11 reads per pool), and Escherichia phage vB_EcoM_G4500 with a total of 12 reads detected in three out of eight datasets (pools 2, 3, and 4; 2–8 reads per pool) (Fig. 4). Two contigs could be assembled from the reads, classified as Escherichia phage PBECO 4 and Synechococcus phage S-CAM7.

### Microbiome

**Fig. 5:**
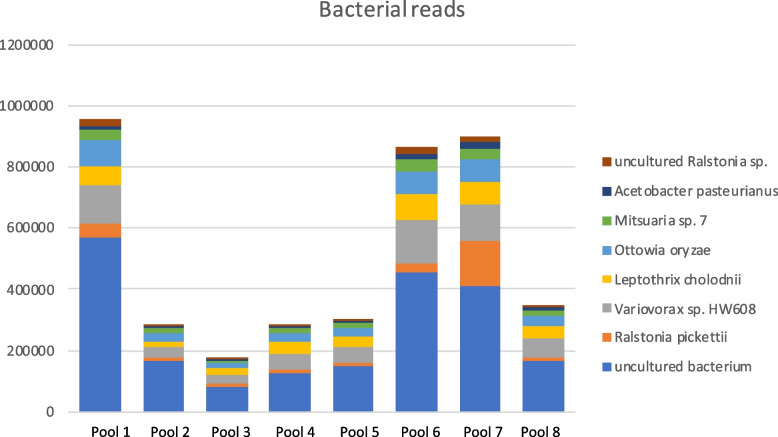
Stacked bar chart showing the number of reads from the eight most abundant bacterial species

Bacterial reads were found in all pools (range 627 031 – 3 241 146 bacterial reads per pool) and the taxonomic classification result showed that the overall microbiome was comparable between the eight pools. The vast majority of the reads were classified, using the nucleotide non redundant (*nt*) NCBI database, into three Gram-negative, bacterial families; *Burkholderiaceae* (approximately 20% of the bacterial reads), *Comamonadaceae* (approximately 9% of the bacterial reads), and *Enterobacteriaceae* (approximately 5% of the bacterial reads). The species that generated the highest number of classified reads were *Variovorax.* sp HW608 (747,316), *Leptothrix cholodnii* (473,624), and *Ralstonia pickettii* (417,795). When blasted, the reads generated *Ralstonia pickettii, Variovorax.* sp, and *Leptothrix cholodnii* hits. About 18% of the bacterial reads were classified as so called “uncultured bacterium” (Fig. 5).

**Fig. 6:**
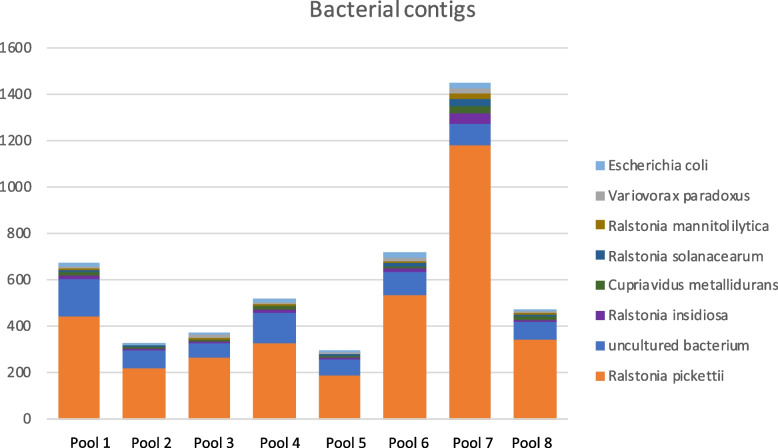
Stacked bar chart showing the number of contigs from the eight most abundant bacterial species

The reads assembled into 758 – 2229 bacterial contigs per pool, in total 10,076 contigs, and the taxonomic assignment of the contigs demonstrated that the seminal microbiome was comparable between the eight pools (Fig. 6). The vast majority of the contigs were classified into three bacterial families, all Gram-negative; *Burkholderiaceae* (approximately 51% of the bacterial contigs), *Comamonadaceae* (approximately 6% of the bacterial contigs) and, *Enterobacteriaceae* (approximately 1% of the bacterial contigs), consistent with the read classification. *Ralstonia* sp. was the most common genus; *Ralstonia pickettii*, *Ralstonia insidiosa*, *Ralstonia solanacearum* and, *Ralstonia mannitolilytica* corresponded to approximately 38% (5053) of the bacterial contigs. *Ralstonia pickettii* was the species that generated the highest number of classified contigs, approximately 29% (4677) of the bacterial contigs. Then, *Variovorax* sp*.* generated approximately 2% (179) of the bacterial contigs, *Cupriavidus* sp*.* (*Cupriavidus metallidurans* previously known as *Ralstonia metallidurans, Cupriavidus taiwanensis*, *Cupriavidus pauculus, Cupriavidus necator*, and *Cupriavidus basilensis)* generated 2% (204) of the bacterial contigs, *Escherichia* sp*.* (*Escherichia coli*) generated < 1% (113) of the bacterial contigs, and *Cutibacterium* sp*.* (*Cutibacterium acnes*) generated < 1% (77) of the bacterial contigs. When blasted, the contigs generated the same hits as Kraken2 generated. About 9% (920) of the bacterial contigs were classified as so called “uncultured bacterium”.

### No detection of antimicrobial resistance genes.

The analysis of ARGs on the bacterial contigs did not reveal the presence of any genes conferring resistance to antibiotics.

### Mycobiome

**Fig. 7:**
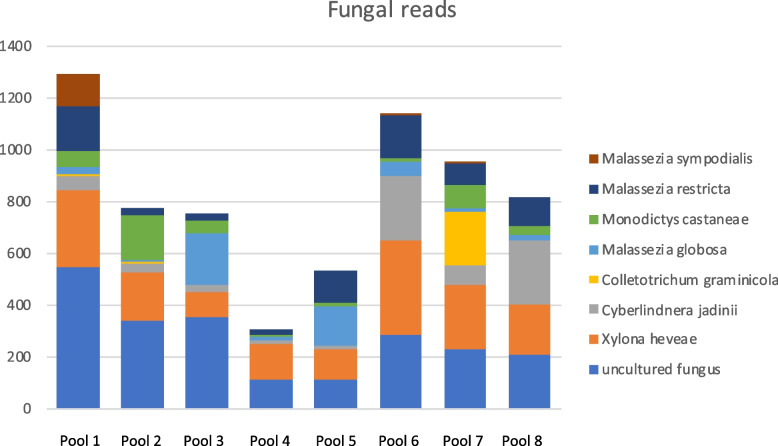
Stacked bar chart showing the number of reads from the eight most abundant fungal species

Fungal reads were detected in all samples (range 1766 – 5630 fungal reads per pool, 25,217 reads in total) but no order represented > 1% of the reads. The most abundant orders were Saccharomycetales, < 1% of the reads (2161), Malasseziales, < 1% of the reads (1907), and Xylonales with < 1% of the reads (1992). About 1% of the fungal reads (2737) were so called “uncultured fungus”. The reads assembled in to 238 contigs (range 20 – 41 across the pools) with reclassification using contig data. *Cyberlindnera jadinii* (order Saccharomycetales) generated approximately 16% [36] of the contigs, *Malassezia globose* (order Malasseziales) generated approximately 10% [24] of the contigs, and *Malassezia restricta*, (order Malasseziales) generated approximately 10% [24] of the contigs (Fig. 7).

## Discussion

The present study aimed to explore the seminal RNA microbiome including the virome of Swedish commercial boar semen from Swedish breeding boars and to screening for APPV. The sequencing results showed that APPV was present in the semen from one boar at the time of sampling. Apart from this, the overall RNA-virome and microbiome of the extended semen contained no obligate pathogens.

The detection of APPV in the AI-dose was unexpected, given that Swedish breeding boars are bred at specific farms with extra high biosecurity, quarantined before being introduced into the stud, and thereafter kept under strict hygiene measures. Nevertheless, the most likely explanation to the detection of APPV is that a transplacentally infected pig, recovered from congenital tremor but being a silent carrier of the virus, was unknowingly accepted as a breeding boar. This theory is strengthened by the fact that APPV was only detected in 1 of 24 semen samples from 2019 and in none of the 100 semen samples from 2021. This finding makes an outbreak of APPV at the breding stud unlikely; generally, when APPV is introduced into a farm, it quickly spreads to the majority of the animals, causing substantial on-farm outbreaks [9, 10, 12, 17, 18, 37]. If APPV had been introduced into the boar stud, we speculate that several boars would shed APPV and that the virus would have been detected in a number of AI-doses during the same period of time.

Although the duration of APPV-excretion in semen post-infection is not established, studies show that transplacentally infected piglets, born with congenital tremor but fully recovered, may shed the virus continuously in semen or preputial fluid until slaughter [12, 17, 18, 38]. Moreover, it is suggested that APPV, similar to other pestivirus, possibly, may cause persistent infections [10, 18, 36, 39]. Taken together, the detection of APPV in one AI-dose has important implications for biosecurity; one ejaculate can be used to produce up to 60 AI doses [1] and controlled, pathogen-free AI semen is crucial for a healthy pig industry.

Phylogenetic analysis suggests the APPV detected in semen is closely related to viruses found both in Europe and the USA. In the analysis, sequences from previous detection of Swedish APPV could not be included as they span the NS3 region. However, previously sequenced Swedish viruses fell into Cluster I and Cluster II, and as such are likely genetically distinct from those recovered here, in line with the findings of others, with a high degree of sequence variation among APPV strains within the same country and region [40].

While screening for APPV was one focus of this study, the high-throughput sequencing approach applied enabled an analysis of the seminal RNA-virome. Posavirus-1, a porcine non-pathogenic faecal associated virus [41] was detected in five of eight sequencing libraries. This is indicative of the expected faecal contamination during semen collection [42]. In addition, a number of phages and retroviruses were unevenly present in the libraries and no homogenous seminal virome could be revealed. The limited virus diversity in the semen supports the hypothesis derived from human studies of a naturally sparse seminal virome [43] and that a low virus diversity in semen is associated with achieving pregnancy [44].

Compared to the seminal virome, the bacterial part of the microbiome was abundant and overall homogenous between the eight semen pools. The majority of the bacterial reads and contigs were classified as Gram-negative bacteria, mainly from the phylum Proteobacteria, where the three most common families were *Burkholderiaceae, Comamonadaceae*, and *Enterobacteriaceae.* This is comparable to the results from the two other available high-throughput sequencing studies on boar semen, where Proteobacteria also was the dominant phylum [7, 8]. The most common bacterial species varied between the studies; *Ralstonia* spp. was the most common species in the present study whereas Zhang et al., 2020 and Gòdia et al., 2020 found *Pseudomonas aeruginosa* and *Bacillus megaterium,* respectively, to be the most common species. Overall, the microbiome from the Swedish boars mainly consisted of apathogenic or opportunistic bacteria and had a lower occurrence of pathogenic bacteria as compared to the results from China [7] and Spain [8]. Literature on the seminal microbiome of healthy boars is limited to these two studies, but in the human literature, the seminal microbiome is stated to be diverse and individual, consisting of aerobic, facultative anaerobic, and strictly anaerobic bacteria, including species considered to be opportunistic pathogens [45–48]. Interestingly, when comparing our results to the results from these high-throughput sequencing studies on semen from healthy men, the results were strikingly similar. Three of the four studies [45, 46, 48] found *Ralstonia* to be the most abundant species in human semen, in line with the results from the present study on breeding boars. This accordance with the results of others is affirmative of our method’s ability to correctly identify viruses and other microbes, although the RNA-virome was the initial focus.

As part of analysing the microbiome, a bioinformatic screening for antibiotic-resistant genes (ARGs) was done. In the datasets from the eight pools, 24 breeding boars in total, no ARGs were detected. Globally, the knowledge of the occurrence of ARGs in commercial boar semen is limited, and only a few studies comprising a low number of individuals are available for comparison. One high-throughput sequencing study on boar semen from Spain (Godia et al., 2020) identified a low occurrence of three different ARGs. In contrast, a study using MALDI-TOF (matrix-assisted laser desorption/ionization time-of-flight) mass spectrometry and API 20 E on boar semen from Romania (Costinar et al., 2021) found more than half of the isolated bacteria to be resistant to gentamycin and penicillin [49]. Although the number of Swedish boars investigated in the current study was limited to 24 individuals, the absence of ARGs is a positive finding for Sweden's policy on antibiotic use.

Healthy AI semen is key for the pig industry and we would like to encourage the porcine breeding industry to consider including screening for APPV in the health program.

## Conclusion

In conclusion, APPV was detected in one semen sample from a boar in a boar stud delivering sperm to most Swedish sow herds. In addition, a sparse and individual RNA virome was identified in the extended semen; mainly consisting of retrovirus, phages, and some stool- associated contaminants. The seminal microbiome, however, was large and more homogenous, being characterized by Gram-negative bacteria from the phylum Proteobacteria and mainly apathogenic or opportunistic bacteria. The proportion of potentially pathogenic bacteria was low, and no ARGs could be detected in the datasets. Overall, these results indicate a good health status among Swedish breeding boars, although, we would like to encourage the boar studs to include screening for APPV in their routine testing of breeding boars.

## Material and methods

### Samples

In total, semen samples from 124 boars were analysed. The samples consisted of extended, ready-to-use AI doses in bags, each containing 80 ml, purchased from one of Svenska Köttföretagen AB.s’ two boar studs.

At the boar stud, the boars were housed in individual pens with a solid floor. Wood shavings was provided as bedding material and haylage was given as coarse fodder. The boars originated from Sweden and Norway. All boars were kept in quarantine for five weeks before being taken in to the boar stud. During quarantine, the boars were tested for *Pasteurella multocida*, *Actinobacillus pleuropneumoniae, Brucella suis,* Methicillin-Resistant *Staphylococcus Aureus,* Aujeszky´s disease virus (SuHV-1), porcine reproductive and respiratory syndrome virus and, swine fever viruses (African and Classical). The boars were also treated with anthelmintics and vaccinated against *Mycoplasma hyopneumoniae*, swine influenza virus, parvovirus, and *Erysopelothrix rhusiopatie*.

Semen was collected in two batches. The first 24 AI doses collected during one week in January 2019 were analyzed by metagenomic sequencing, and 100 AI doses collected during three weeks in October 2021 were screened by qPCR for the presence of the APPV genome. In both cases, one AI dose represented one individual boar. All doses were delivered in an insulated box, the day after the collection (first 24 doses) or on the day of collection, to the Swedish University of Agricultural Sciences in Uppsala.

A calculation of the sample size to estimate the apparent prevalence of APPV positive breeding boars was done; Estimated true proportion: 0.01, desired precision of estimate: 0.05, confidence level: 0.95, and population size: 400, generating a sample size of 16 individuals for the specified inputs (Sergeant, ESG, 2018. Epitools Epidemiological Calculators. Ausvet. Available at: http://epitools.ausvet.com.au).

### Samples preparation for metagenomic sequencing

The AI-semen doses were transferred into tubes and centrifuged at 4 °C, 12 000 RFC for 3 h. The supernatant was removed, transferred to clean tubes, and stored overnight at -80 °C. In the morning, RNA extraction was performed. From each sample, 170 µL supernatant was thawed on ice and treated with 8 µL (2U/ul) of TURBO™ DNase (Invitrogen, Thermo Fisher Scientific, Pittsburgh, PA, USA), 2 µL DNase I (Thermo Fisher Scientific, Pittsburgh, PA, USA), and then incubated at 37 °C for 30 min. The total volume of 180 µL was treated with 5 µL Ambion® RNase Cocktail™ (Sigma-Aldrich®, Darmstadt, Germany) and incubated at room temperature for 5 min. The RNA was extracted using Trizol® approach as described by Stenberg et al. (2020) and the GeneJET RNA kit (Thermo Fisher Scientific, Waltham, MA, USA) according to the manufacturers’ instructions. An on-column DNase digestion treatment was performed during the RNA extraction, using a RNase-Free DNase Set (QIAGEN GmbH, Hilden, Germany). The concentration of the purified RNA was quantified on a Qubit® 2.0 Fluorometer using the Qubit RNA HS assay kit (Thermo Fisher Scientific, Paisley, UK). The samples were normalized based on the RNA concentration, and semen from three boars of the same breed were pooled, generating 8 pools for sequencing.

### Library preparation and sequencing

The sequencing libraries were prepared using a Trio RNA-Seq Library Preparation Kit with the Custom AnyDeplete for targeted pig (*Sus scrofa domesticus*) genome depletion (NuGEN Technologies, San Carlos, CA, USA). Following preparation, the libraries were normalized to 5 ng/ µL based on the concentration as measured by TapeStation (Agilent, Santa Clara, CA, USA) and Qubit® 2.0 Fluorometer (Thermo Fisher Scientific, Paisley, UK). RNase-free water was used for dilution of the libraries that were stored at -20 °C before being sequenced.

A paired-end 150 bp read length sequencing was performed on the NovaSeq 6000 system using a SP flow cell and the v1 sequencing chemistry (Illumina, San Diego, CA, USA). A sequencing library for the phage PhiX was included as 1% spike-in in the sequencing run. The sequencing was performed by the SNP&SEQ Technology Platform, National Genomics Infrastructure (NGI) Sweden at the Science for Life Laboratory in Uppsala. The quality of the produced dataset was assessed using the FastQC software [50].

### Bioinformatics analysis of the metagenomics datasets

A NextFlow pipeline [51] including the following steps: read quality control and trimming using FASTP version 0.19.5 [52], removing of the host reads by mapping on *Sus scrofa domesticus* genome using Bowtie2 version 2.3.5.1 [53], and de novo assembly of the reads using MEGAHIT version 1.2.9 [54] was used to perform the analyses on each dataset. The pipeline is fully available online (https://github.com/jhayer/nf-metavir). The taxonomic classification of the reads was performed using Kraken 2 version 2.0.8-beta [55] run against the nucleotide non-redundant (*nt*) NCBI database. For the taxonomic classification of the assembled contigs, Kraken2 was run against the non-redundant protein database (*nr*) [56]. The produced reports were visualised using Pavian [57]. In addition to the NextFlow pipeline, a specific alignment against the APPV genome (NC_030653.1 Atypical porcine pestivirus 1 isolate Bavaria S5/9 polyprotein gene, complete cds) was performed on all datasets using Bowtie2 version 2.3.5.1 [53].

### Analysis of Antimicrobial Resistance Genes (ARGs)

All assembled contigs that were classified as bacterial by Kraken2 were extracted using KrakenTools (https://github.com/jenniferlu717/KrakenTools) and submitted to an ARGs screening using two different methods: the NCBI AMRFinderPlus (version 3.10.45, database release 2022–10-11), and the Resistance Gene Identifier (RGI, version 6.0.0) using the CARD database (release September 2022).

### Samples preparation for qPCR-screening

The one hundred semen samples collected in 2021 were screened by PCR for the presence of APPV. On the day of collection, the samples were dispensed into tubes and stored at -80 °C. The subsequent RNA-extractions were performed at the National Veterinary Institute, Uppsala, using a standardised method for semen RNA-extraction with an extraction robot and the EZ1Virus Mini kit v2.0 (Qiagen, Hilden, Germany). Prior to the extraction, the semen was diluted 1:4 in TE-buffer (pH 8.0).

### APPV genome verification and qPCR (quantitative reverse transcription-PCR)

The APPV-genome verification and qPCR-screening were done on individual AI doses using an APPV-specific qPCR protocol based on the QuantiTect Probe RT-PCR kit (Qiagen, Hilden, Germany) as described by Postel et al., [10] with a primer-pair and probe targeting the NS3-encoding region of the APPV genome. A plasmid expressing the APPV NS3 encoding region, kindly provided by Postel et al., [10], was used as a positive control. The assay was run with duplicate samples, under standard conditions on a Bio-Rad CFX96™ Real-time system in a C1000 Touch™ thermal cycler (Bio-Rad, Hercules, CA, USA). The qPCR-analysis was done on the individual AI doses.

### Phylogenetic analysis

All available Posavirus-1 and APPV sequences were mined from GenBank and aligned using MAFFT and the E-INS-i algorithm, implemented in Geneious prime (https://www.geneious.com). As the contigs recovered spanned different parts of the genome, we took an approach of removing the regions of the alignments between contigs. Specifically, to capture all three Posavirus contigs, we concatenated three alignment blocks ranging from position 1072–3987 (non-structural region spanning the RNA helicase) relative to NC_023637, for a final alignment length of 1446 bp. To capture both Atypical Porcine Pestivirus contigs, we concatenated the 5’UTR and a region spanning the end of Erns and first half of E1, for a final alignment length of 692 bp. Maximum likelihood trees were estimated using PhyML, implementing the best nucleotide substitution model as determined by smart model selection, integrated into PhyML (Guindon et al., [58]). Support values were calculated using aBayes, a bayesian approach for rapid support estimation (Anisimova et al., [59]).

## Data Availability

The datasets generated and/or analysed during the current study are available in the PRJEB51770 repository at the European Nucleotide Archive. [https://www.ebi.ac.uk/ena/browser/view/PRJEB51770, accession number: SAMEA13468669 — SAMEA13468676].
